# Photoreceptor Cells Constitutively Express IL-35 and Promote Ocular Immune Privilege

**DOI:** 10.3390/ijms23158156

**Published:** 2022-07-24

**Authors:** Cheng-Rong Yu, Manoj Kumar Yadav, Minkyung Kang, Yingyos Jittayasothorn, Lijin Dong, Charles E. Egwuagu

**Affiliations:** 1 Molecular Immunology Section, Laboratory of Immunology, National Eye Institute (NEI), National Institutes of Health (NIH), Bethesda, MD 20892, USA; yuc@nei.nih.gov (C.-R.Y.); manoj.yadav2@nih.gov (M.K.Y.); wookandchan2@gmail.com (M.K.); 2 Immunoregulation Section, Laboratory of Immunology, National Eye Institute (NEI), National Institutes of Health (NIH), Bethesda, MD 20892, USA; yingyos.jittayasothorn@nih.gov; 3 Genetic Engineering Core, National Eye Institute (NEI), National Institutes of Health (NIH), Bethesda, MD 20892, USA; dongl@nei.nih.gov

**Keywords:** uveitis, experimental autoimmune uveitis (EAU), interleukin 35 (IL-35), immunosuppressive cytokines, Th1/Th17 cells, photoreceptors, retina

## Abstract

Interleukin-27 is constitutively secreted by microglia in the retina or brain, and upregulation of IL-27 during neuroinflammation suppresses encephalomyelitis and autoimmune uveitis. However, while IL-35 is structurally and functionally similar to IL-27, the intrinsic roles of IL-35 in CNS tissues are unknown. Thus, we generated IL-35/YFP-knock-in reporter mice (p35-KI) and demonstrated that photoreceptor neurons constitutively secrete IL-35, which might protect the retina from persistent low-grade inflammation that can impair photoreceptor functions. Furthermore, the p35-KI mouse, which is hemizygous at the *il12a* locus, develops more severe uveitis because of reduced IL-35 expression. Interestingly, onset and exacerbation of uveitis in p35-KI mice caused by extravasation of proinflammatory Th1/Th17 lymphocytes into the retina were preceded by a dramatic decrease of IL-35, attributable to massive death of photoreceptor cells. Thus, while inflammation-induced death of photoreceptors and loss of protective effects of IL-35 exacerbated uveitis, our data also suggest that constitutive production of IL-35 in the retina might have housekeeping functions that promote sterilization immunity in the neuroretina and maintain ocular immune privilege.

## 1. Introduction

The interleukin 12 (IL-12) family of cytokines has profound influence on lymphocyte developmental decisions and plays critical roles in regulating immunity by determining lymphocyte subsets recruited to sites of inflammation [1,2]. The family is comprised of IL-12, IL-23, IL-27, IL-35 and IL-39 and belongs to the type 1 family of hematopoietic cytokines [1,3,4,5]. Each member is comprised of an α-subunit with a helical structure similar to type 1 cytokine, IL-6 and a β-subunit structurally related to the soluble IL-6 receptor (IL-6Rα) [1,3]. Chain-pairing promiscuity is a distinctive feature that accounts for distinct effects of IL-12 cytokines on host immunity [1,2]. Some members (IL-12, IL-23 and IL-39) are proinflammatory, while IL-35 suppresses inflammation and IL-27 can be immunosuppressive or proinflammatory depending on the physiological context. Nevertheless, IL-12 family cytokines regulate immunity during autoimmune diseases including central nervous system (CNS) diseases, multiple sclerosis (MS) and uveitis.

CNS inflammatory diseases present unique challenges, as neurons in the retina and brain are largely terminally differentiated cells, which cannot be replaced if lost to injury induced by cytotoxic cytokines secreted by inflammatory cells. Thus, the need to eliminate a pathogen is as important as avoiding exuberant inflammatory responses that damage or kill the neurons required to maintain functional integrity of the CNS [6,7,8]. Studies in mouse models of uveitis and MS have established involvement of IL-12, IL-23, IL-27 and IL-35 in initiating or regulating encephalomyelitis and ocular inflammatory diseases. Thus, targeted deletion or neutralization of T cells that produce IL-12 and/or IL-23 or cell therapy based on administering regulatory B cells (Breg) or T cells (Treg) that produce IL-27 or IL-35 has been found to be effective in suppressing experimental autoimmune uveitis (EAU) and experimental autoimmune encephalomyelitis (EAE) that serve as mouse models of uveitis and MS, respectively [9,10].

IL-35 (p35/Ebi3) is structurally similar to IL-27 (p28/Ebi3), and both heterodimeric cytokines are composed of an alpha-chain (p28, p35) and a common beta-chain (Ebi3) [1,2]. Their α/β subunits are secreted independently and associate extracellularly to form the non-covalently linked heterodimeric IL-27 or IL-35 by poorly understood mechanisms. They are in contrast to the proinflammatory IL-12 and IL-23, which are secreted as disulfide-linked heterodimers. IL-27 is constitutively expressed in the retina, upregulated during intraocular inflammation (uveitis) by retinal microglial cells, and mitigates uveitis by antagonizing pathogenic Th17 cells that mediate the disease [11]. Other reports found increased IL-27 levels in cerebrospinal fluid (CSF) and active plaques of MS patients, and the local secretion of IL-27 is thought to induce activation of regulatory responses that promote resolution of chronic inflammation in the CNS and to initiate tissue repair [12]. These results are in line with a recent report that a novel IL-27-producing B-1a population suppresses uveitis and encephalomyelitis by proliferating and sustaining IL-27 production in the retina, brain and spinal cord of mice [9]. On the other hand, microglia initiate protective immunity in the CNS by promoting vascularization and infiltration of immune cells into the retina [13,14]. However, the IL-27 produced by activated microglia cells amplifies microglia-induced neuroinflammation during sustained CNS inflammation by enhancing expression of proinflammatory cytokines, nitric oxide and antigen presentation functions of the microglial [12,15].

Despite the structural and functional similarities between IL-27 and IL-35, it is not known whether IL-35 is also constitutively expressed by neurons and glial cells and if it has intrinsic function in the normal retina or during CNS autoimmune diseases. In this study, we generated IL-35 reporter mice and used them to track IL-35 expression in cells within the normal retina or during intraocular inflammation. Evidence provided here suggests that IL-35 may have an intrinsic role in sterilization immunity in the retina in maintaining ocular immune privilege.

## 2. Results

### 2.1. Generation and Characterization of il12a-yfp Reporter Mice

**Figure 1:**
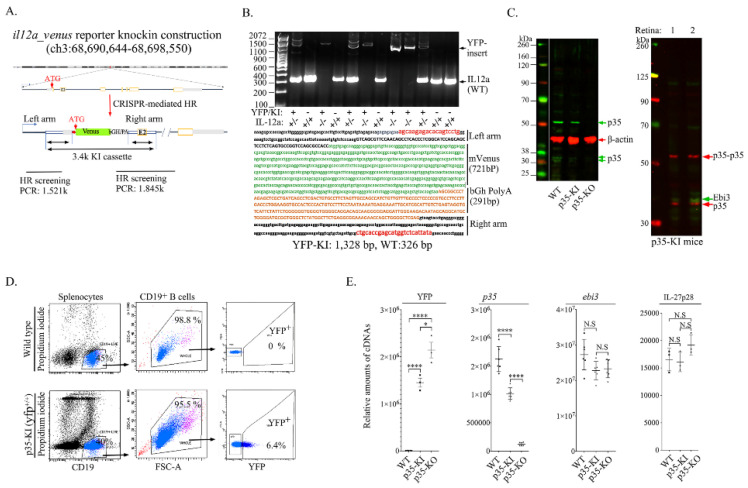
Generation and characterization of IL-35 reporter mouse strain. (**A**) Schematic of the DNA and cDNA constructs used to genetically engineer the *il-12a-venus* yellow fluorescent protein (yfp) knock-in reporter mouse by CRISPR/Cas9-mediated homologous recombination at the C57BL/6J *il-12a* locus. ATG in exon 1 indicates integration site of the guide RNA (gRNA), which is 5′ proximal to the Venus cDNA and bGHPA (bovine growth hormone polyadenylation signal), as shown. The complete 3814 bp *il-12a-yfp* knock-in allele cassette was inserted between the left and right homologous arms, and PCR primers used to validate homologous recombination (HR) are shown. (**B**) Tail DNA genotype analysis showing PCR products used to identify mice with successful homologous recombination. Wild-type control mice (WT) express the IL-12p35 protein but not yfp (*il12^+/+^*); mice expressing yfp from both chromosomes (*il12a*^yfp/yfp^) do not express IL-12p35 (*il12^−/−^*) and are henceforth referred to as IL-12p35 knock-KO (p35-KO); mice expressing yfp only from one chromosome (*il12a*^yfp/−^) express IL-12p35 and yfp proteins (*il12^+/^*^−^) and are henceforth referred to as IL-12p35 knock-in (p35-KI). Details of the entire construct delineate various components of the construct and expected PCR products derived from analysis of tail DNA of WT, p35-KI or p35-KO mice. (**C**) Western blot analysis of whole cell protein extracts of spleen cells from WT, p35-KI or p35-KO mice. (**D**) B cells isolated from the spleen of WT or p35-KI mice were sorted for CD19^+^ B cells, and cell surface FACS analysis established that ~6.4% CD19^+^ cells in the spleen of p35-KI mice express yfp. (**E**) Total RNA isolated from CD19^+^ B cells of the WT, p35-KI or p35-KO mice were analyzed by qPCR using primers specific to mouse to determine the relative abundance of b-actin, IL-12p35 or ebi3 mRNA transcripts. Data represent at least 3 independent experiments. * *p* < 0.05, **** *p* < 0.0001; N.S, no significant differences.

IL-27 (IL-27p28/Ebi3) and IL-35 (IL-12p35/Ebi3) are immunosuppressive cytokines produced by myeloid and lymphoid cells and have been implicated in the suppression of autoimmune diseases [1,9,11,16]. Surprisingly, IL-27 is constitutively and also inducibly expressed in the retina and contributes to the suppression of ocular inflammatory disease or uveitis [17,18]. In this study, we generated an IL-35 reporter mouse to investigate whether IL-35 is expressed by resident cells within the retina. The IL-12p35 reporter knock-in allele carries a yellow fluorescent protein (*il12a*-*yfp*-KI) and was generated by inserting an yfp-gene cassette (venus-bGH/PA) in front of exon 1 of the mouse *il12a* gene through CRISPR-Cas9-mediated homologous recombination. Thus, the YFP expression cassette is integrated upstream of the coding sequence of the IL-35 gene so that the KI allele also serves as a knockout (KO) allele for the target gene (Figure 1). We confirmed that the cassette was properly inserted into the *il12a* locus by PCR and sequencing of the entire genomic region (Figure 1A,B, Supplementary Figure S1). F0 founders in SJ.B6 (H1 hybrid) background homozygous for the *il12a*^fyp+/fyp+^ (*il12a*^Y/Y^) were identified and show no detectible expression of IL-35 (Figure 1B). F1 founders were back-crossed to the C57bl/6J background for several generations to become congenic with C57BL/6J. Taken together, the WT C57BL/6J mouse has two copies of *il12a* and no *yfp*; p35-KO has two copies of *yfp* and no *il12a*; p35-KI strain contains one copy of *il12a* and one copy of *yfp*. Western blot analysis showed that the hemizygous *il12a*^fyp+/fyp−^ (*il12a*^Y/−^) mice express yfp and p35 proteins, while the il12a^Y/Y^ mice do not express p35 (Figure 1C). It is of note that the p35 protein has 10 methionine and 7 cysteine residues and exhibits a propensity to form homodimers and high order aggregates, which accounts for the detection of multiple p35 aggregates [10,19,20]. Henceforth, we refer to *il12a*^fyp+/fyp+^ (*il12a*^Y/Y^) mice as p35-KO and *il12a*^fyp+/fyp−^ (*il12a*^Y/−^) mice as p35-knock-in or p35-KI. As IL-35 expression has mainly been reported in a relatively rare population of regulatory B-cell (i35-Bregs) lymphocytes, it was necessary to rule out the possibility that yfp expression might be promiscuously expressed. We isolated and sorted CD19^+^ B cells from the spleen of WT or p35-KI mice, and FACS analysis of the cells shows that only ~6.4% of the B cells in p35-KI were YFP positive (Figure 1D), which is consistent with previous reports that less than 7.8% of B cells express IL-35 upon activation [10]. In contrast to WT B cells that were similarly activated, YFP^+^ cells were not detectable in the WT mice (Figure 1D). Analysis of the sorted CD19^+^ B cells by qPCR confirmed the expression of p35 by the p35-KI but not the p35-KO B cells (Figure 1E). On the other hand, there was no difference in *ebi3* expression levels (Figure 1E). Taken together, the p35-KI mouse is generated to have the reporter cassette replacing the p35 open reading frame (ORF), while the p35-KO mouse missing both copies of *il12a* serves as a knockout (KO) allele for the target gene. Hence, the results presented above established fidelity of p35-KI as a bona fide reporter mouse strain for use in identifying cells that constitutively express IL-35 in the neuroretina and tracing their cell-fate during inflammation. Importantly, the p35-KI and p35-KO do not exhibit any observable physiological abnormalities.

### 2.2. IL-35 Is Constitutively Expressed by Resident Cells in the Mouse Retina

**Figure 2:**
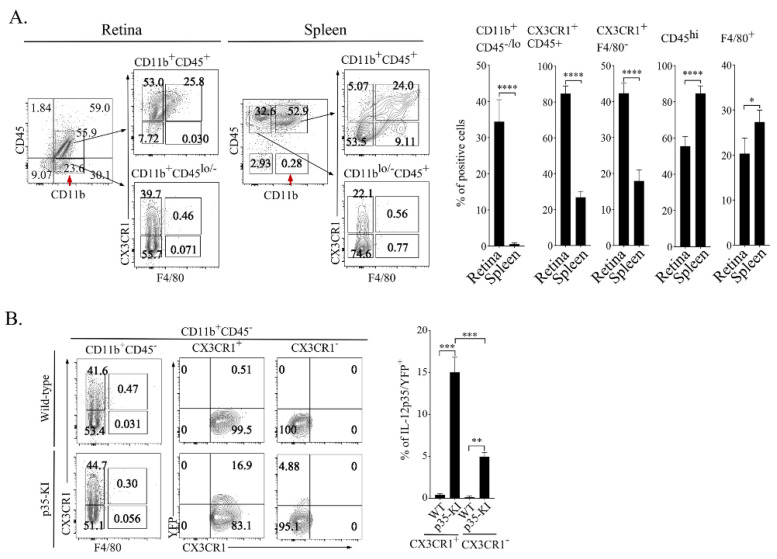
Retinal microglia constitutively express IL-35. (**A**) Characterization of microglial cells in the retina and spleen by FACS analysis. Cells were gated using monoclonal antibodies specific to mouse CD45, CD11b and Cx3CR1. Representative flow cytometry plots showing percent CD11b^+^CD45^−/lo^, Cx3CR1^+^CD45^+^, Cx3CR1^+^F4/80^−^, CD45^hi^ and F4/80^+^ cells (**left panels**) and bar graphs show percentage of exhibiting these immunophenotypes in the retina or spleen (**right panels**). (**B**) Single cell preparations of cells isolated from the retina of WT or p35-KI mice were analyzed and gated for CD45^lo^ (myeloid cells) and cells expressing YFP and p35. Numbers in quadrants represent the percentage of microglial cells (CD11b^+^CD45^lo^CX3CR1^+^) and other myeloid cell types (CD11b^+^CD45^lo^CX3CR1^−^). Data represent at least 3 independent experiments. * *p* < 0.05; ** *p* < 0.01; *** *p* < 0.001; **** *p* < 0.0001.

Secretion of IL-27 in the retina by microglia is associated with the suppression of uveitis and neuroprotective effectives that promote the survival of photoreceptors [11,15,17,21]. Microglia in the retina are characterized by the CD11b^+^CD45^lo^CX3CR1^+^F4/80^−^ phenotype [22,23], and given the structural/functional similarities between IL-27 and IL-35, we considered the microglia as the logical cell type in the retina that might express IL-35. We isolated cells from the retina and spleen of wild-type C57BL/6j and p35-KI mice, sorted for cells that express CD45 and CD11b cell surface proteins that serve as markers of all hematopoietic and myeloid cells. FACS analysis detected significant elevation of cells expressing the CD11b^+^CD45^lo^CX3CR1^+^F4/80^−^ microglia cell phenotype (Figure 2A). Although CD11b^+^CD45^lo^CX3CR1^+^F4/80^−^ is a distinctive phenotypic marker of retina microglia cells in the retina, monocytes and macrophages also express some of these cell surface markers. Nonetheless, analysis of WT and p35-KI mouse retina revealed a significantly high percentage of YFP^+^ CD11b^+^CD45^lo^CX3CR1^+^F4/80^−^ cells in the p35-KI mouse retina (Figure 2B), providing suggestive evidence that IL-35 is expressed in the retina by the microglia cells.

### 2.3. IL-35 Is Constitutively Expressed in the Photoreceptor Layer of Wild-Type Mouse Retina

**Figure 3:**
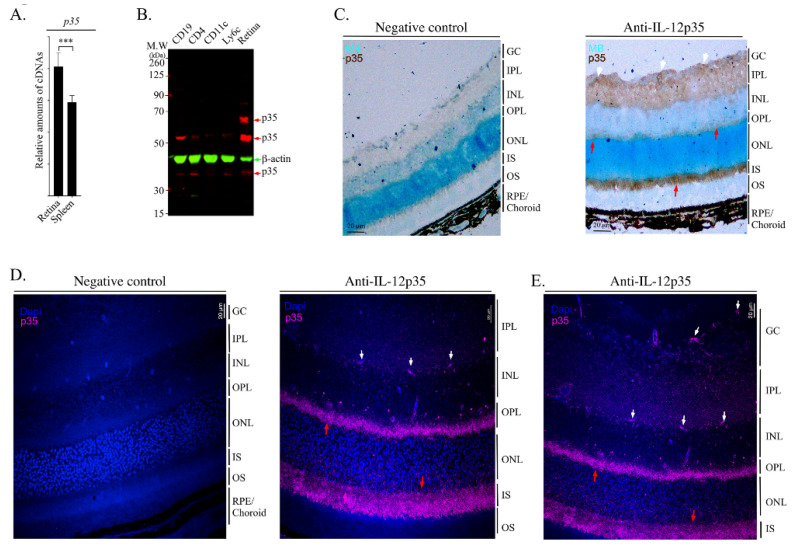
IL-35 is highly expressed in retinal rod and cone photoreceptors. (**A**) qPCR analysis of p35, ebi3 IL-10 mRNA transcripts in the retina and spleen of WT mice. (**B**) Western blot analysis of whole cell extracts prepared from sorted CD4^+^ T cells, CD19^+^ B cells, dendritic cells (CD11c) and monocytes (Ly6C). Antibodies used were specific to mouse p35 or β-actin. (**C**) Immunohistochemistry (IHC) analyses of frozen eye sections were performed with or without primary mouse IL-12p35 antibodies and goat anti-mouse (fab’2) secondary antibody. Slides were then stained with a DAB kit and counter-stained with methyl blue. IL-12p35-expressing cells, dark brown; microglia, white arrows; photoreceptor layers, red arrows. (**D**,**E**) Detection of IL-12p35-expressing cells by immunohistochemistry and confocal microscopy. Paraffin-fixed whole eye sections were subjected to antigen-retrieval, blocked in mouse blocking solution, incubated without or with primary rabbit IL-12p35 antibody and then goat anti-rabbit conjugated with AF647 antibody. Far-red magenta color indicates IL-12p35 expression; red arrow, photoreceptor layers; white arrow, microglial cells. GC, ganglion cell layer; IPL, inner plexiform layer; INL, inner nuclear layer; OPL outer plexiform layer, ONL, outer nuclear layer; IS, inner section; OS, outer segment; RPE/choroid, retinal pigmented epithelial and choroid. Data represent at least 3 independent experiments. *** *p* < 0.001.

We isolated RNA from the retina or spleen of C57BL/6J mice, qPCR analysis shows significant expression of IL-12p35 mRNA transcripts in the retina (Figure 3A), and this was confirmed at the protein level (Figure 3B). As shown by Western blot analysis, the level of p35 protein is substantially higher in the retina compared to lymphoid or myeloid cell types (Figure 3B), indicating that cells in the retina constitutively express the p35 protein in the absence of ocular inflammation. We next investigated the resident retina cell types that produce IL-35 with particular focus on microglia, which are the main producers of IL-27 in the retina [11]. For these analyses, mice were extensively perfused with saline solution to remove blood from the central retinal artery and the choriocapillaris to ensure that residual blood cells in these blood vessels would not confound our analysis. The eyes were enucleated, fixed in paraffin and sectioned or stored as frozen whole eye sections as described in the Methods section. Immunohistochemistry (IHC) and DAB (3, 3′-diaminobenzidine) staining of frozen whole eye sections localized expression of the p35 protein to microglia in the ganglion cell layer and inner plexiform layer (IPL), as well as at the photoreceptor cell inner segment and outer plexiform layer (OPL) (Figure 3C). Interestingly, while we detected punctate staining of microglia-expressing p35 cells in the ganglion cell layer (white arrows), most of the IL-35-producing cells (red arrows) were in the photoreceptor inner segment and OPL (Figure 3C). We verified and confirmed this surprising and unexpected finding by IHC/confocal microscopy using highly sensitive fluorescence-labelled monoclonal antibodies (Figure 3D,E). The confocal image also detects IL-35 expression in the OPL that includes synapses from the INL and photoreceptor cell inner segments, as well as synapses between rod cell endings or cone cell branched foot plates in the ONL, suggesting the IL-35 expression might also derive from other synapses, such as those between the dendrites of horizontal cells. Together, these results suggest that photoreceptor cells might be the major producers of IL-35 in the retina while IL-35 expression by microglia is restricted to the inner retina.

### 2.4. p35-KI Mice Develop Severe Uveitis in Mice

**Figure 4:**
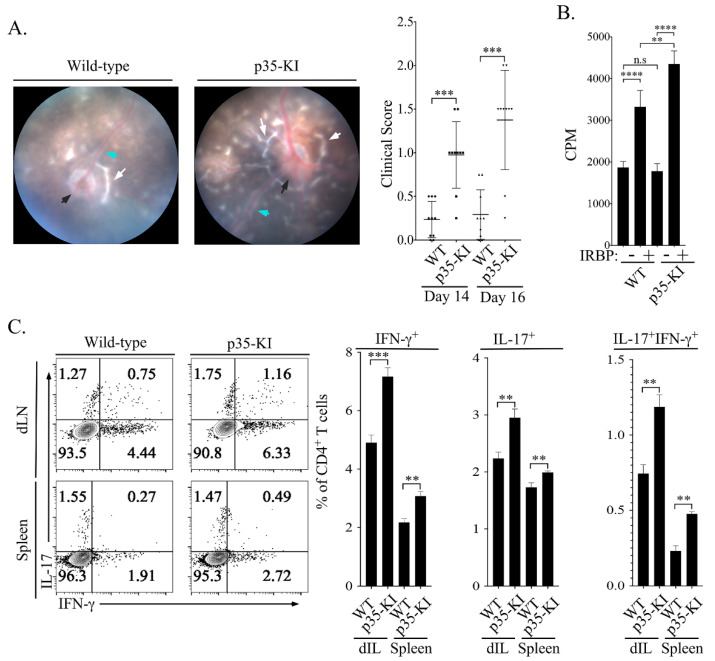
p35-KI mice develop severe experimental autoimmune uveitis (EAU). (**A**) EAU was induced in WT C57BL/6J or p35-KI mice by immunization with IRBP_651-670_ peptide in CFA (*n* = 12), and disease progression was analyzed by fundoscopy. Black arrow indicates inflammation with blurred optic disc margins and enlarged juxtapupillary areas; blue arrows indicate retinal vasculitis; white arrows indicate yellow-whitish retinal and choroidal infiltrates. Clinical scores and assessment of disease severity were based on changes at the optic nerve disc or retinal vessels and retinal and choroidal infiltrates. (**B**) Draining LN cells (dLN) isolated from C57BL/6J or p35-KI mice with EAU, stimulated in vitro in the absence or presence of IRBP_651-670_, and proliferation of the cells was assessed by the [^3^H]-thymidine incorporation assay as described in Methods section. Data are presented as mean values of CPM of five replicate cultures. (**C**) dLN and spleen cells from C57BL/6J or p35-KI EAU mice and analyzed by the intracellular cytokine expression assay. Numbers in quadrants indicate percentage of CD4^+^ T cells expressing IFN-γ and/or IL-17. Results represent 3 independent studies. ** *p* < 0.01; *** *p* < 0.001; **** *p* < 0.0001.

We next investigated the physiological role of constitutive expression of IL-35 in the retina, particularly during intraocular inflammation. We induced experimental autoimmune uveitis (EAU) in WT C57BL/6J and p35-KI mice by active immunization with IRBP_651-670_ peptide in complete Freund’s adjuvant (CFA). EAU is characterized by the recruitment of proinflammatory lymphocytes of the Th1 and/or Th17 subsets into the retina, and the intraocular inflammation or uveitis is generally manifested between post-immunization (p.i.) day 14 and day 22 [11,24,25]. We monitored progression and severity of uveitis during this time period by fundoscopy, a non-invasive procedure that allows visualization of the fundus of the eye (retina, macula, optic disc, fovea and blood vessels) using an ophthalmoscope or fundoscopy. Fundoscopic images obtained on day 21 p.i. show classical features of uveitis, which include papillitis, blurred optic disc margins and enlarged juxtapupillary areas (black arrows); retinal vasculitis (blue arrows); yellow-whitish retinal and choroidal infiltrates (white arrows) (Figure 4A). On the other hand, the fundus images reveal a more severe disease in the eyes of p35-KI mice, showing significantly higher clinical scores compared to the eyes of WT mice (Figure 4A; right panel). Because EAU is mediated by IL-17 and/or IFN-γ-producing T cells, we investigated whether the development of severe uveitis in p35-KI mice derived from aberrant expansion of IRBP-specific pathogenic T lymphocytes and inflammatory responses induced by Th17 and/or Th1 cells. We isolated lymphocytes from EAU mice 21 days after EAU induction and re-stimulated the uveitogenic T cells with IRBP_651-670_ and the ^3^H-thymidine incorporation assay shows that T cells derived from p35-KI mice exhibit higher proliferative responses compared to T cells of WT mice (Figure 4B). In addition, intracellular cytokine staining analysis shows a significant increase of IFN-γ- and -IL-17-producing Th1 and Th17 cells in the p35-KI mice (Figure 4C). The expansion of Th17 cells expressing IL-17 and IFN-γ (DP-Th17) in draining lymph nodes (dLN) and spleen of p35-KI mice is of clinical relevance, as the DP-Th17 population is implicated in severe inflammatory diseases including uveitis [11,20], Crohn’s disease [26], candidiasis [27] and multiple sclerosis [28].

### 2.5. Downregulation of IL-35 Expression Correlates with Increased Severity of EAU

**Figure 5:**
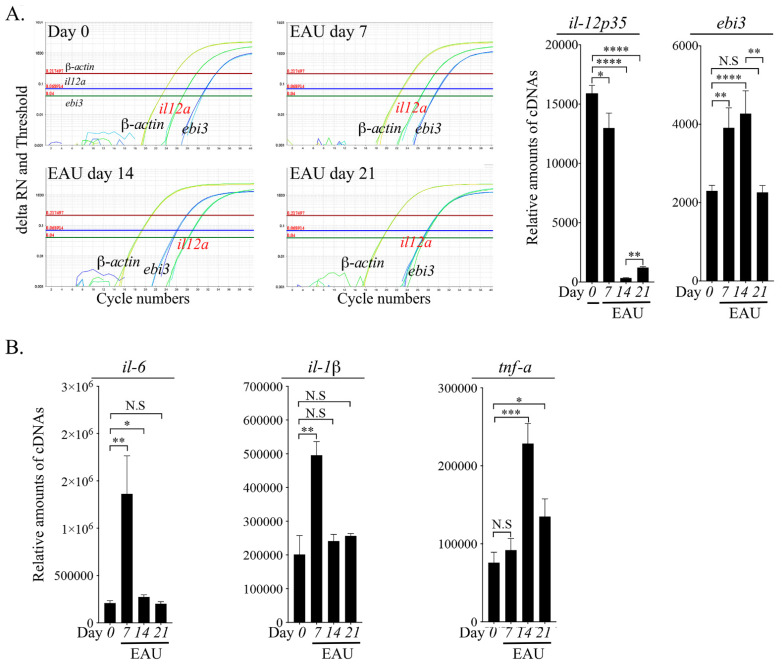
Decrease of IL-35 at EAU onset correlates with increase of inflammatory cytokines. WT C57BL/6J mice were immunized with IRBP_651-670_ in CFA, and mice were sacrificed at several time points (day 0, day 7, day 14 and day 21) after EAU induction. After perfusing with PBS, retina was isolated, RNA was immediately isolated, and cDNA was subjected to qPCR analysis. (**A**,**B**) Histograms show relative abundance of IL-35 subunit mRNA transcripts *IL-12p35* and *ebi3* (**A**) and mRNA transcripts for the proinflammatory cytokines IL-6, IL-1β and TNF-α (**B**). Results represent 3 independent studies. * *p* < 0.05; ** *p* < 0.01; *** *p* < 0.001; **** *p* < 0.0001.

The p35 suppresses inflammation in part through regulation of cell cycle inhibitory proteins including cyclin D1, cyclin E, cyclin A and cyclin-dependent kinase inhibitor p27^kip1^ [29]. In this study, we found that T cells derived from p35-KI mice with EAU exhibit higher proliferative responses to the IRBP autoantigen (Figure 4B), and this observation is inconsistent with previous findings that IL-12p35 suppresses lymphocyte proliferation by inhibiting cell cycle regulatory proteins including cyclin D1, cyclin E, cyclin A and cyclin-dependent kinase inhibitor p27^kip1^ [29]. To understand the mechanistic explanation for this apparent inconsistency, we isolated cells from the retina of p35-KI mice at various time points during the course of EAU. Surprisingly, analysis of RNA derived from the retina cells detected relatively high levels of p35 mRNA transcripts at day zero to day seven when the mice exhibited no evidence of uveitis (Figure 5A). However, by day seven, we began to observe a progressive reduction of p35 mRNA levels followed by a precipitous diminution in the level of *p35* expression by day 14, which corresponds to the peak of EAU (Figure 5A). On the other hand, *ebi3* mRNA transcript increased from day zero, reaching a peak level on day 14 and then declining to its basal level by day 21 (Figure 5A). These results suggest that expression of p35 is downregulated during ocular inflammation with modest change in the expression of the Ebi3 subunit shared by IL-35 and IL-27. We further show that downregulation of p35 expression at the peak of EAU correlates temporally with the transcriptional upregulation of *il-6, tnf-α* and *il-1β* required for the expression of cytokines that mediate proinflammatory responses (Figure 5B). Because IL-35 contributes to mechanisms that suppress CNS inflammatory diseases [10,20,29,30], we believe that severe uveitis in the p35-KI mouse strain is derived in part from significant reduction of IL-35 caused by inflammation-induced loss of the IL-35-producing photoreceptor cells. Thus, IL-35 produced by photoreceptor cells may contribute to the maintenance of ocular immune privilege and sterile immunity in the retina, while transient downregulation in response to pathogenic or toxic stimuli may be required to allow development of protective immunity in the eye.

## 3. Discussion

The neuroretina is a functional unit of the central nervous system (CNS) that converts photons to electrical signals, which are then transduced to the brain to create visual perception [24]. It is comprised of photosensitive photoreceptors (rods and cones), retina interneurons (horizontal cells, bipolar cells, amacrine cells) and output neurons (ganglion cells), as well as glial cells (astrocytes, Müller and microglia cells) [24]. Like the brain, the retina is an avascular immune-privileged tissue that contains resident retinal cells that secrete anti-inflammatory cytokines or neuropeptides and contribute to immunological sequestration of the retina [31]. Recent reports have identified IL-35 and IL-27 as playing important roles in maintaining CNS immune privilege and suppressing neuroinflammation by propagating inhibitory signals that convert conventional lymphocytes into regulatory cells that inhibit the inflammatory responses of encephalitogenic and uveitogenic lymphocytes.

Although IL-27 is constitutively produced by microglia in the retina and brain, it is not known whether regulation of immunity during autoimmune or infectious diseases derives from production of IL-35 by inflammatory cells that enter the CNS following the breakdown of the blood–brain barrier or blood–retina barrier. In this study, we generated an IL-35 reporter mouse model that has allowed us to provide direct evidence that IL-35 is constitutively expressed in the neuroretina. In contrast to IL-27, which is expressed primarily by retina microglia and localized to the ganglion cell layer [11,18], we show here that IL-35 expression is localized to the outer plexiform layer and photoreceptor outer segment and presumably produced by photoreceptor cells in the outer retina. Thus, the distinct spatial localization of IL-27 and IL-35 expression suggests that these immune suppressive cytokines may have distinct and/or overlapping physiological functions in the developing retina or during inflammation. Moreover, expression of IL-27 and IL-35 contiguous to where microglia and photoreceptors reside in the retina, respectively, suggests that these glial (microglia) and specialized neurons (rods and cones) might perform unique functions in the retina that require IL-27 and IL-35. Expression of IL-27 by microglia has been shown to have a double-edged-sword effect on immunity, as the activated M1-like microglia required to initiate protective immunity in the CNS [9,11,32] can also exhibit deleterious activities during sustained inflammation or noxious insults as underscored by the suppression of ocular inflammation in mice lacking microglia [13,14]. On the other hand, the M2-like microglia is associated with tissue repair and recovery from injury, and IL-35 regulates the M1/M2 macrophage ratio and promotes microglial M2 polarization and single nucleotide polymorphemic changes in *il-12a* (SNP rs2243115) correlates with susceptibility to Vogt–Koyanagi–Harada syndrome (VKH), a severe panuveitis with acute onset [33,34,35]. These observations illustrate the complex and not well understood functions of IL-27- or IL-35-producing cells in the retina or intraocular immunity.

Uveitis is a diverse group of potentially sight-threatening intraocular inflammatory diseases that includes Bechet’s disease, birdshot retinochoroidopathy, Vogt–Koyanagi–Harada disease, sympathetic ophthalmia and ocular sarcoidosis [36]. Although the etiology of uveitis is not fully understood, proinflammatory T lymphocytes of the Th1 and Th17 subset that induce breakdown of the blood–ocular barrier and promote infiltration of inflammatory cells into the retina are implicated [24,37]. Most of our current understanding of the immunopathogenesis of uveitis has come from studies of EAU, a disease in mice that shares essential pathologic features of human uveitis and is considered the quintessential model of human uveitis [37,38,39]. We show here that EAU is exacerbated in the p35-KI and exhibited a clinical disease score that is >4-folds higher than the counterpart WT control mice. Lymphocyte proliferation and intracellular cytokine expression assays revealed significant expansion of Th1 and Th17 cells in the dLN and spleen of the p35-KI mice. The expansion of Th1/Th17 cells and exacerbation of uveitis in the p35-KI mice were unexpected and paradoxical in view of the fact that IL-35 cytokine or IL-12p35 as well as the adoptive transfer of IL-35-producing regulatory cells (i35-Bregs) suppress uveitis in mice [10,20].

Although the two immunosuppressive members of the IL-12 family cytokines, IL-27 and IL-35, are structural similar and are constitutively expressed in the retina, they appear to have different functions in the retina. Depending on the physiological context, IL-27 can be a proinflammatory or immunosuppressive cytokine. To understand the effects of IL-27 produced by microglia, it is important to appreciate that the diverse microglia subpopulations in the retina can exhibit distinct features, and whether IL-27 induces inflammatory or neuroprotective responses may depend on the phenotype of the microglia or if IL-27 is produced during the acute or chronic phase of the disease [13,14,40]. While a primary function of microglia in EAU is to initiate the disease by promoting the breakdown of the blood–retina barrier and extravasation of inflammatory cells into the retina, the IL-27 produced by microglia confers protection against uveitis by suppressing the expansion of pathogenic Th17 cells that mediate EAU [11,13]. In contrast, IL-35 appears to be strictly immunosuppressive, and the light-sensing and photon-transducing photoreceptor cells are major producers of IL-35 in the retina. Interestingly, time course analysis of IL-35 production in the retina during EAU revealed expression of the highest level of p35 prior to initiation of EAU, followed by a precipitous decline due to onset of the disease, indicating a strong correlation between the decrease of IL-35 expression and the massive loss of photoreceptor cells that occurs during EAU. Moreover, the downregulation of IL-35 expression at the peak of EAU also coincided with the upregulation of proinflammatory cytokines such as TNF-α and IL-6 that initiate and promote pathology in uveitis. Given that IL-27 and IL-35 share the common Ebi3 subunit, this raises the question of whether an increase of the physiological level of IL-27 would suppress IL-35 production because of competition for the Ebi3 in the retina. It is therefore of note that each IL-12 family cytokine is characterized by a unique alpha (α) subunit (p19, p28 or p35) that heterodimerizes with one of the beta (β) subunits (p40 or Ebi3), and each of these cytokines that include IL-12 (p35 and p40), IL-23 (p19 and p40), IL-27 (p28 and Ebi3) and IL-35 (p35 and Ebi3) is independently produced and regulated [1,2]. Moreover, the two β subunits, Ebi3 and p40, are abundantly expressed in many cell types and competition to heterodimerize with Ebi3 or p40 has not been shown to influence the level of these cytokines in vivo. Despite sharing the common Ebi3 subunit, IL-27 and IL-35 are not coordinately regulated, as they can have different functions depending on the physiological context, and they are often produced by distinct cell types. For example, IL-27 is produced mainly by innate B-1a cells in the peritoneal cavity and human umbilical blood, while IL-35 is produced mainly by B-2 cells in the spleen [9,10]. In this study, we have shown that IL-35 is produced predominantly by cells in the photoreceptor layers, while previous reports indicate that IL-27 is produced mainly by microglia [11,18]. Thus, the pattern of expression of IL-35 and IL-27 in different layers of the retina may account for their different functions, with IL-27 initiating protective immunity to pathogens by promoting extravasation of inflammatory cells into the retina, while IL-35 may function to restrain the unbridled pathogenic inflammation that causes chronic uveitis.

In summary, this study reveals that the anti-inflammatory cytokine IL-35 is constitutively produced by cells in the retina, including the photoreceptor rod cells that mediate peripheral vision at night or low illumination conditions and the cone cells that function in bright light and are required for color vision. We also show that proinflammatory lymphocytes such as Th1 and Th17 cells that breakdown the blood–ocular barrier and infiltrate the retina during intraocular inflammatory diseases target and destroy photoreceptor cells and thereby compromise the capacity to produce sufficient IL-35 required to confer protection of the neuroretina from ocular pathology. Our data thus provide suggestive evidence that IL-35 might contribute to mechanisms that maintain immune privilege in the retina.

## 4. Methods and Materials

### 4.1. Mice

Six- to eight-week-old C57BL/6J mice were purchased from Jackson Laboratory (Jackson Laboratory, Bar Harbor, ME). The p35-KI and p35-KO mouse strains were generated and housed in NIH mouse facility. Mice used for all experiments were 6–8 weeks old and sex-matched per institutional guideline. The mice were maintained and treated in accordance with National Eye Institute (NEI) and NIH Animal Care and Use Committee guidelines (study # EY000262-19 and EY000372-14). All animal care and experimentation conformed to National Institutes of Health (NIH) guidelines, and the experimental protocol was approved under NIH/NEI Animal Study Protocol (ASP) # NEI-597.

### 4.2. il12a-Venus Knock-in through CRISPR-Mediated Homologous Recombination

The p35-KI/KO allele was created using CRISPR-mediated homologous recombination directly in zygotes of the SJL.B6 (F1) hybrid strain with a double strand DNA targeting construct as the recombination template [41]. Guide RNAs (gRNA, for SpCas9, PAM = NGG) were selected for the intended recombination based on their relative positions to the target sequence and ranking by the online gRNA selection tool (www.CRISPRscan.org, 6 June 2019). gRNAs were synthesized with T7 in vitro transcription as described and further tested for their efficiencies of in vitro cleavage and in-cell culture indel mutagenesis activities [42]. For the in vitro cleavage assay, genomic PCR product containing the target sites of selected gRNAs was incubated with SpCas9 protein (NEB, New England Biolabs, Ipswich, MA, USA) by following manufacturer’s suggested protocol and analyzed on 2% agarose gel stained with ethidium bromide (not shown). Guide RNAs were further tested for their efficiencies inducing indels at target sites in an immortalized mouse embryonic fibroblast (MEF) cell line engineered to carry a tet-inducible Cas9 expression cassette (unpublished, not shown). Upon confirmation of efficient target cleavage activity in MEF cells, gRNAs were mixed with SpCas9 protein (PNA Bio, Thousand Oaks, CA, USA) along with the targeting construct as described above. The targeting construct was designed to insert the YFP expression cassette upstream of the endogenous coding sequence of IL-35 gene so that the KI allele also serves as a knockout allele of the target gene (Figure 1A). The gRNAs used to generate the KI allele are *il-12p35*_gR1 GATTGACACATGC TGGAC and *il-12p35*_gR2 CAGCGTGATTGACACATGC. F0 founder mice were screened for the presence of the homologous recombination event by PCR flowing with sequencing confirmation. Mice carrying the desired KI/KO allele were backcrossed to C57BL/6J mice for several generations.

### 4.3. Experimental Autoimmune Uveitis (EAU)

EAU was induced by active immunization of mice with IRBP_651-670_-peptide (300 µg/mouse) in a 0.2 mL emulsion (1:1 *v*/*v* with complete Freund’s adjuvant (CFA) containing 2.5 mg/mL *Mycobacterium tuberculosis* strain H37RA (2.5 mg/mL)) sub-cutaneous, as described previously [43]. Mice also received *Bordetella pertussis* toxin (1 µg/mouse) by intraperitoneal (i.p.) injection in 100 µL of RPMI 1640 medium containing 1.0% normal mouse serum on the same day of immunization. For each study, 8–12 mice (6–8 weeks old) were used per group, and they were matched by age and sex. Clinical disease was established and scored by fundoscopy, as described previously [10]. Eyes were examined for disease severity using binocular microscope with coaxial illumination. Eyes for histology were enucleated, fixed in 10% buffered formalin and serially sectioned in the vertical pupillary–optic nerve plane. Sections were stained with hematoxylin and eosin.

### 4.4. Fundoscopy

Fundus examinations were performed following administration of anesthesia (intraperitoneal injection of ketamine (1.4 mg/mouse) and xylazine (0.12 mg/mouse)), and the pupil was dilated by topical administration of 1% tropicamide ophthalmic solution (Alcon Inc., Fort Worth, TX, USA). Fundus image was captured using Micron III retinal imaging microscope (Phoenix Research Labs, Pleasanton, CA, USA) for small rodents or a modified Karl Storz veterinary otoendoscope coupled with a Nikon D90 (St. Louis, MO, USA) digital camera, as described [44,45]. Evaluation of fundus photographs was conducted without knowledge of the mouse identity by masked observers. At least 6 images (2 posterior central retinal view, 4 peripheral retinal views) were taken from each eye by positioning the endoscope and viewing from superior, inferior, lateral and medial fields. The clinical grading system for retinal inflammation was as established [46,47].

### 4.5. Retina Cells Isolation

Mice were anesthetized and perfused with 1× PBS and retina isolated from enucleated eyes in culture medium (RPMI 1640) under a dissecting microscope. Briefly, the eye was cut along the limbus and the lens and cornea were carefully removed. Then, the retina was peeled off and the attached optic nerve was removed before digesting the freshly isolated retina with collagenase (1 mg/mL) in RPMI medium containing 10 µg/mL DNase (Sigma-Aldrich, St. Louis, MO, USA) for 2 h at 37 °C. During incubation, the cells were pipetted intermittently every 30 min, and the digestion reactions were quenched with 5–10-folds volume 10% FBS in RPMI 1640 medium. The cells were washed twice in complete RPMI-1640 medium, and cells were counted using the Vi-Cell XR cell viability analyzer (Beckman Coulter, Indianapolis, IN, USA).

### 4.6. Fluorescence-Activated Cell Sorting (FACS) Analysis

Cells from the draining LN (dLN) and spleen were re-activated in vitro with IRBP_651-670_ and assessed for IL-17 and IFN-γ expression by intracellular cytokine analysis. For FACS analysis, the retinal cells and the activated lymphocytes were stained with monoclonal antibodies conjugated with fluorescent dyes. Each tube of stained cells was color-compensated and dead cells or aggregated cells were excluded during analysis. Quadrant gates were set using isotype controls with less than 0.5% background. For intracellular cytokine analysis, PMA (50 ng/mL; Sigma-Aldrich), ionomycin (250 ng/mL; Sigma-Aldrich) and brefeldin A (BD bioscience, San Jose, CA, USA) were added for the last 4 h of cell culture, and cells were fixed and permeabilized using a Cytofix/Cytoperm kit (BD Biosciences) according to the manufacturer’s instructions. Antibodies used were monoclonal Abs specific to CD4, IL-17, CD27 or CD43 (BD bioscience), and Dead Cell Staining Kit was from Invitrogen. For surface FACS analysis of retinal cells, the fresh retinal cells were immediately stained with Fixable Viability Dye (ViaKrome 808, Cat # C36628, Beckman Coulter, Indianapolis, IN, USA), CD11b (clone M1/70) (Cat # 101230, BioLegend), IA/IE (clone M5/114.15.2, Cat # 557000, BD), F4/80 (clone BM8 Cat # 123122, BioLegend), Ly6c (clone AL-21 Cat # 560596, BD), Cx3CR1 (clone SA011F11 Cat # 149123, BioLegend) and CD45 (clone 30-F11 Cat# 563981, BD). FACS analysis was performed on a MACSQuant analyzer (Miltenyi Biotec), and data were analyzed using Flowjo V10.8.

### 4.7. Immunohistochemistry (IHC) and Confocal Microscope

Immunohistochemistry (IHC) and confocal microscope experiments were performed as previously described [48]. Frozen or paraffin fixed whole eye sections were stained with Anti-IL-12p35 mouse IgG1 (cat: mAB1570) and rabbit (cat: ab131039) monoclonal antibodies from R&D. Antigen retrieval for the frozen section was carried out by heating at 80 °C for 30. For paraffin section, antigen retrieval was carried out by autoclave at 120 °C for 5 min in Tris-EDTA buffer ph9.0. For sections incubated with HRP secondary antibodies, endogenous peroxidase was blocked using 3% hydrogen peroxide, followed by incubation with 10% goat serum, 1% BSA and 0.01% Triton in PBS. DAB staining was performed with Sk4100 vector DAB kit, and sections were counter-stained with methyl blue. For confocal microscopy, fluorescence-labeled slides were counter-stained with DAPI and visualized using Zeiss Axio Cam 5 brightfield/Zeiss 880 confocal microscope. Same gain/exposure/laser power settings were used for negative and positive control.

### 4.8. Quantitative RT PCR (qPCR) Analyses

Total RNA was extracted using TRizol reagent, and RNA samples were digested with RNase-free DNase I as described [49]. All RNAs were subjected to quality assessment by analysis of 18S and 28S ribosomal RNA expression using Agilent RNA 600 Nano Reagent Kit on the Agilent 2100 Bioanalyzer system (Agilent Technologies, Santa Clara, CA, USA). cDNA synthesis was performed using superscript III reverse transcriptase and oligo(dT)12–16 as recommended by the manufacturer (Invitrogen-Thomas Scientific, Carlsbad, CA, USA). cDNA preparations were normalized to β-actin. Real-time PCR using TaqMan primers/probes and quantification of mRNA transcripts were as previously described [49].

### 4.9. Western Blot Analysis

Whole cell lysates were prepared as previously described [49]. Protein extracts (20µg/lane) were fractionated on 4–12% gradient NuPAGE Bis-Tris gels (Invitrogen, Carlsbad, CA, USA) under reduced condition. Blots were probed with antibodies specific to mouse β-actin (sc-47778), and EBI3 (sc-166158) was purchased from Santa Cruz Biotechnology (Santa Cruz, CA, USA). Anti-IL-12a (IL-12p35) was purchased from Abcam (ab131039 clone EP5737). All fluorescent-labeled secondary antibodies were purchased from the LI-COR system (LI-COR Biosciences, Lincoln, NE, USA). Membranes incubated with the secondary antibody were used as negative controls. Western membranes were scanned in The Odyssey Imaging System (LI-COR Biosciences, Lincoln, NE, USA).

### 4.10. CD19^+^ B Cells Isolation

Primary mouse CD19^+^ B cells were isolated from spleens of wild-type and p35-KI mice by using CD19-microbeads (130-052-201, Miltenyi Biotec Inc., Street Auburn, CA, USA) as described [50].

### 4.11. Proliferation Assay

Uveitogenic T cells were re-stimulated in vitro in the presence or absence of IRBP peptide for 72 h. CD19^+^ B cells were stimulated with anti-CD40 Abs (10 µg/mL) and anti-IgM Abs (5 µg/mL) in the presence or absence of IL-27. Cells were pulsed with ^3^H-thymidine (0.5 µCi/10µL/well) for the last 24 h in culture. Presented data are mean CPM ± S.E.M. of responses of 5 replicate cultures.

### 4.12. Statistical Analysis

Statistical analyses were performed by independent two-tailed Student’s *t* test. Statistical analysis was performed by Student’s *t* test (two-tailed). Asterisks denote *p* value (* *p* < 0.05; ** *p* < 0.01; *** *p* < 0.001; **** *p* < 0.0001).

## Data Availability

Not applicable.

## Supplementary Materials

The following supporting information can be downloaded at: https://www.mdpi.com/article/10.3390/ijms23158156/s1.
