# Supplementary material for: Photoreceptor Cells Constitutively Express IL-35 and Promote Ocular Immune Privilege
Source: Int J Mol Sci. 2022 Jul 24;23(15):8156. doi: 10.3390/ijms23158156 (PMC9351654; doi:10.3390/ijms23158156)
Supplement: Supplementary file 1 [file ijms-23-08156-s001.zip › ijms-1804158-SI.pdf]

S1A.

Locations of PCR primers.

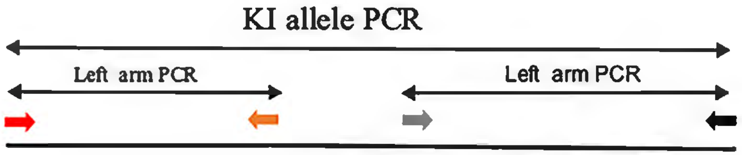

Sizes of PCR products.

PCR product of the KI allele (3.814k) with primer set IL12a E2-sc-F (red arrow) and IL12a E2-right-R (dark arrow) covering the KI cassette and the HR junctions are sequenced and confirmed to be correct on two F0 mice: CH12-20 and CH12-42

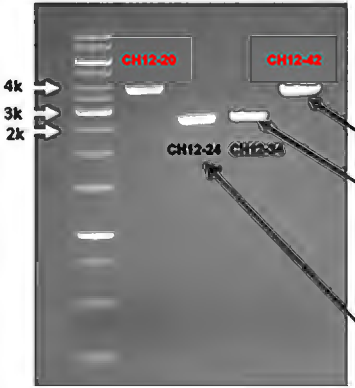

IL12a E2-sc-F: ctgctgagtaagtgctcactgatctct

IL12a E2-right-R: tggagcggagcttatgttgggatagc

3.8k KI allele

2.8k wt allele

IL12aE2 venus-R1521: ggtggtgcagatcagcttcagggtcagc

IL12a E2 venus-F: acctgagctaccagtccaagctgagc

Mouse founder names

S1B.

Left Arm PCRs

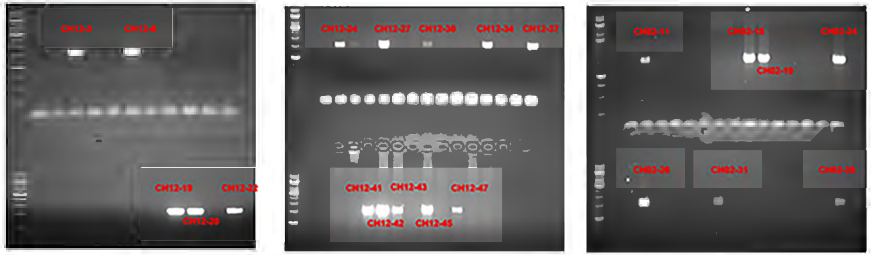

Primer set: IL12a E2-sc-F + IL12aE2 venus-R1521 (PCR amplicon=1521bp) PCR enzyme: GXL

Total 22 pups are positive on left arm: CH12- 3, 6, 19, 20, 22, 24, 27, 30, 34, 37, 41, 42, 43, 45, 47, CH02- 11, 18, 19, 24, 26, 31, 39

Right Arm PCRs

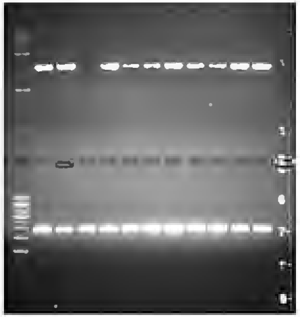

All F0 positive for the left arm HR event are also positive for the right arm HR event, except CH12-19:

CH12- 3, 6, 19, 20, 22, 24, 27, 30, 34, 37, 41, 42, 43, 45, 47

CH02- 11, 18, 19, 24, 26, 31, 39

Primer set: IL12a E2 venus-F+ IL12a E2-right-R, 1845bp PCR enzyme: GXL
